# The Effects of *N*-Butyl-1-(4-dimethylamino)phenyl-1,2,3,4-tetrahydro-****β****-carboline-3-carboxamide against *Leishmania amazonensis* Are Mediated by Mitochondrial Dysfunction

**DOI:** 10.1155/2013/874367

**Published:** 2013-06-13

**Authors:** Hélito Volpato, Vânia Cristina Desoti, Juliana Cogo, Manuela Ribeiro Panice, Maria Helena Sarragiotto, Sueli de Oliveira Silva, Tânia Ueda-Nakamura, Celso Vataru Nakamura

**Affiliations:** ^1^Programa de Pós-Graduação em Ciências Biológicas, Universidade Estadual de Maringá, Avenida Colombo 5790, PR 87020-900 Maringá, Brazil; ^2^Programa de Pós-Graduação em Ciências Farmacêuticas, Universidade Estadual de Maringá, Avenida Colombo 5790, PR 87020-900 Maringá, Brazil; ^3^Departamento de Química, Universidade Estadual de Maringá, Avenida Colombo 5790, PR 87020-900 Maringá, Brazil; ^4^Programa de Pós-Graduação em Ciências Biológicas, Laboratório de Inovação Tecnológica no Desenvolvimento de Fármacos e Cosméticos, Bloco B-08, Universidade Estadual de Maringá, Avenida Colombo 5790, PR 87020-900 Maringá, Brazil

## Abstract

Leishmaniasis is a disease that affects millions of people worldwide. The drugs that are available for the treatment of this infection exhibit high toxicity and various side effects. Several studies have focused on the development of new chemotherapeutic agents that are less toxic and more effective against trypanosomatids. We investigated the effects of *N*-butyl-1-(4-dimethylamino)phenyl-1,2,3,4-tetrahydro-*β*-carboline-3-carboxamide (**C4**) and its possible targets against *L. amazonensis*. The results showed morphological and ultrastructural alterations, depolarization of the mitochondrial membrane, the loss of cell membrane integrity, and an increase in the formation of mitochondrial superoxide anions in *L. amazonensis* treated with **C4**. Our results indicate that **C4** is a selective antileishmanial agent, and its effects appear to be mediated by mitochondrial dysfunction.

## 1. Introduction 

Leishmaniasis is a disease caused by the protozoa of the genus *Leishmania*, which belongs to the order Kinetoplastida, family Trypanosomatidae. An estimated 12 million people are affected by this disease [1], with 2 million new cases worldwide [2] reported in 88 countries and four continents [3]. This disease has two clinical forms, visceral and cutaneous leishmaniasis.

The currently available first-line treatments are pentavalent antimonials [4]. These drugs have high toxicity and adverse side effects [5]. Amphotericin B and pentamidine are second-line therapies but are associated with long-term treatment, limited effectiveness, significant side effects, and toxicity [6–8]. Consequently, an urgent need exists to discover new drugs that are effective against leishmaniasis.

**Figure 1:**
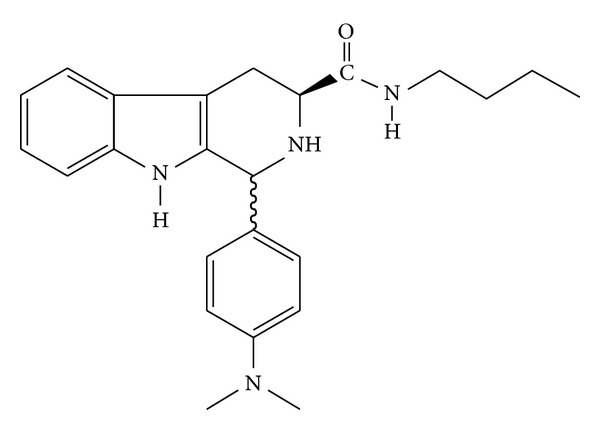
Chemical structure of *N*-butyl-1-(4-dimethylamino)phenyl-1,2,3,4-tetrahydro-*β*-carboline-3-carboxamide (**C4**).

Several studies are being conducted to find new antileishmanial compounds. Carbolines comprise a class of compounds that have an alkaloid indole nucleus and hydrogenated six-member pyridine ring [9]. They are distributed throughout nature in many living beings, including vegetables, fungi, animals, and even human fluids [10]. Carbolines can be divided into three groups: fully aromatic, dihydrocarbolinic, and tetrahydro-*β*-carboline [11]. Interestingly, natural and synthetic *β*-carbolines are well known to possess several biological properties. For example, our group recently reported the effective trypanocidal activity of the synthetic compound *N*-butyl-1-(4-dimethylamino)phenyl-1,2,3,4-tetrahydro-*β*-carboline-3-carboxamide (**C4**; Figure 1) against *Trypanosoma cruzi* [12, 13].

The mechanism of action of *β*-carboline compounds involves changes in DNA and inhibition of the respiratory chain in epimastigote forms of *T. cruzi* [10]. Although the mode of biological action of this compound in different cell types is not fully understood, previous studies have shown that the *β*-carboline ring in the molecule might be deposited in base pairs of DNA, thus contributing to its biological activity [14, 15] or inducing apoptosis [16].

Considering the low efficacy of drugs against leishmaniasis and previous studies of the effects of *β*-carboline compounds on *T. cruzi*, we investigated the potential effect of the synthetic compound *N*-butyl-1-(4-dimethylamino)phenyl-1,2,3,4-tetrahydro-*β*-carboline-3-carboxamide (**C4**) against *L. amazonensis* and its possible targets in this protozoan.

## 2. Materials and Methods

### 2.1. Chemicals

Antimycin A, carbonyl cyanide m-chlorophenylhydrazone (CCCP), digitonin, dimethylsulfoxide (DMSO), and rhodamine 123 (Rh123) were purchased from Sigma-Aldrich (St. Louis, MO, USA). Fetal bovine serum (FBS) and RPMI-1640 were obtained from Invitrogen (Grand Island, NY, USA). 3,8-Phenanthridinediamine-5-(6-triphenylphosphoniumhexyl)-5,6-dihydro-6-phenyl (MitoSOX) and propidium iodide (PI) were obtained from Invitrogen (Eugene, OR, USA). All of the other reagents were of analytical grade.

### 2.2. Synthesis of **C4** Compound

**C4** was synthesized as previously described by Valdez et al. [12].

### 2.3. Preparation of Drugs

 The **C4** compound was prepared in DMSO. All of the groups, including controls, were tested at final concentrations of less than 1% DMSO, a concentration that was found to not affect parasite or mammalian cells (data not shown).

### 2.4. Parasites and Cell Culture

*L. amazonensis *(strain WHOM/BR/75/JOSEFA) was originally isolated from a patient with diffuse cutaneous leishmaniasis by C.A. Cuba-Cuba (Universidade de Brasília, Brazil). Promastigotes were cultured in Warren's medium (brain heart infusion, hemin, and folic acid; pH 7.0) supplemented with 10% inactivated FBS at 25°C. Axenic amastigotes were cultured in Schneider's medium (Sigma, St. Louis, MO, USA; pH 4.6) supplemented with 20% FBS at 32°C.

Macrophages (J774G8) were maintained in tissue flasks with RPMI-1640 (pH 7.2), added with sodium bicarbonate and L-glutamine (As annex), and supplemented with 10% FBS at 37°C in a 5% CO_2_ atmosphere.

### 2.5. Antiproliferative Assay

Promastigote and axenic amastigote forms in the logarithmic phase (1 × 10^6^ cells/mL) were cultured on a 24-well plate in Warren's and Schneider's media, respectively, supplemented with FBS in the presence or absence of 2.56, 12.8, 25.6, 128.0, and 256.0 *μ*M of **C4**. The activity against promastigotes and axenic amastigotes was evaluated after 72 h of incubation. The results are expressed as a percentage, and the IC_50_ (i.e., 50% inhibitory concentration) was determined after-incubation [17].

### 2.6. Cytotoxicity Assay in Macrophage Cells

Cytotoxicity was evaluated in J774G8 macrophage cells. A suspension of 5 × 10^5^ cells/mL was cultured in RPMI-1640 medium supplemented with 10% FBS and added to each well in 96-well microplates. The plates were incubated at 37°C in a 5% CO_2_-air mixture to obtain the confluent growth of the cells. After 24 h, the compound was added to each well at increasing concentrations (160.0, 320.0, 640.0, and 1,280.0 *μ*M), and the plates were incubated for 48 h in a 5% CO_2_-air mixture at 37°C. After treatment, the medium was removed and washed with phosphate-buffered saline (PBS), and 50 *μ*L of MTT (3-[4,5-dimethylthiazol-2-yl]-2,5-diphenyltetrazolium bromide formazan; 2 mg/mL) was added to each well for 4 h in a 5% CO_2_-air mixture at 37°C. DMSO (50 *μ*L) was then added, and the plates were homogenized. Absorbance was read in a 96-well plate reader (BIO-TEK Power Wave XS spectrophotometer) at 492 nm. The percentage of viable cells was calculated relative to controls, consisting of cells cultured in medium alone, according to CC_50_ values (i.e., 50% cytotoxicity concentration). The CC_50_ was determined by logarithm regression analysis.

### 2.7. Scanning Electron Microscopy

 The promastigote forms of the parasite in the logarithmic phase (1 × 10^6^ cells/mL) in the presence or absence of 16.0 and 103.0 *μ*M of **C4** for 48 h were washed with 0.01 M PBS and fixed in 2.5% glutaraldehyde in 0.1 M sodium cacodylate buffer for 2 h at room temperature. The parasites were adhered on poly-l-lysine-coated coverslips, dehydrated in different concentrations of ethanol, critical-point dried with CO_2_, sputter coated with gold, and observed in a Shimadzu SS-550 scanning electron microscope [18].

### 2.8. Transmission Electron Microscopy

 The promastigote forms of the parasite in the logarithmic-phase (1 × 10^6^ cells/mL) in the presence or absence of 16.0 and 103.0 *μ*M of **C4** for 48 h were harvested by centrifugation and fixed in 2.5% glutaraldehyde in 0.1 M sodium cacodylate buffer. The cells were then post-fixed in a solution that contained 1% osmium tetroxide and 0.8% potassium ferrocyanide at room temperature for 60 min, dehydrated in different concentrations of acetone, and embedded in Epon resin. Thin sections were stained with uranyl acetate and lead citrate and examined in a JM 1400 JEOL transmission electron microscope [19].

### 2.9. Mitochondrial Membrane Potential and Cell Membrane Integrity Assay

The promastigote forms of the parasite in the logarithmic phase (5 × 10^6^ cells/mL) in the presence or absence of 16.0 and 103.0 *μ*M of **C4** for 24 h were harvested and washed with PBS. The parasites were then washed and incubated at 37°C with Rh 123 (5 *μ*g/mL for 15 min) to evaluate mitochondrial membrane potential (ΔΨm) and PI (0.2 *μ*g/mL for 10 min) to verify possible alterations in cell membrane integrity. CCCP (100 *μ*M) and digitonin (40 *μ*M) were used as positive controls for mitochondria membrane potential alterations and cell membrane alterations, respectively. The material was kept on ice until analysis. The mean fluorescence intensity of the cells was analyzed by flow cytometry using FACSCalibur and CellQuest software. A total of 10,000 events were acquired in the region that was previously established as the one that corresponded to the parasites [20].

### 2.10. Fluorimetric Detection of Mitochondrial-Derived O_2_^∙−^

Promastigote forms of the parasite in the logarithmic phase (2 × 10^7^ cells/mL) were harvested and washed with Krebs-Henseleit (KH) solution buffer that contained 15 mM NaHCO_3_, 5 mM KCl, 120 mM NaCl, 0.7 mM Na_2_HPO_4_, and 1.5 mM NaH_2_PO_4_ (pH 7.3). The cells were loaded with 5 *μ*M MitoSOX reagent. The parasites were incubated for 10 min at room temperature (25°C) and protected from light. After incubation with MitoSOX reagent, the parasites were washed twice with KH buffer and treated or not with 16.0, 103.0, and 205.0 *μ*M of **C4**. Antimycin A (10 *μ*M), a stimulus that is known to induce superoxide anion (O_2_^∙−^) production by mitochondria, was used as a positive control. MitoSOX detection was performed using black 96-well plates for 3 h. Fluorescence was measured using a fluorescence microplate reader (Victor X3; PerkinElmer) at 194 *λ*ex = 510 nm and *λ*em = 580 nm [21].

### 2.11. Statistical Analysis

The data shown in the graphs are expressed as the mean ± standard error of at least three independent experiments. The data were analyzed using analysis of variance (ANOVA). Significant differences among means were identified using Tukey post hoc test. Values of *P* ≤ 0.05 were considered statistically significant. The statistical analyses were performed using Statistica software.

## 3. Results

### 3.1. Antileishmanial Activity

The treatment of the parasites with **C4** dose dependently inhibited the growth of the promastigote and axenic amastigote forms of *L. amazonensis*. The inhibition percentages of the parasites and concentrations that corresponded to 50% (IC_50_) and 90% (IC_90_) of growth inhibition of the promastigotes were calculated by plotting the concentration *versus* percentage growth inhibition using linear regression after directly counting free-living parasites in a Neubauer chamber. The IC_50_ and IC_90_ in promastigotes were 16.0 ± 2.28 *μ*M and 103.0 ± 20.17 *μ*M, respectively. In axenic amastigotes, the IC_50_ and IC_90_ were 16.3 ± 2.38 *μ*M and 118.0 ± 45 *μ*M, respectively (Table 1).

### 3.2. Cytotoxicity Assay

The cytotoxic effect (50% cytotoxic concentration [CC_50_]) of **C4** in J774G8 macrophages after 48 h of treatment was 722.0 ± 45 *μ*M (Table 1). The toxicity in macrophages was compared with activity against the promastigote and axenic amastigote forms, yielding the selectivity index (SI). **C4** was more selective against the promastigote and axenic amastigote forms than against mammalian cells, with SIs of 45.1 and 44.2, respectively (Table 1).

### 3.3. Scanning and Transmission Electron Microscopy

**Figure 2:**
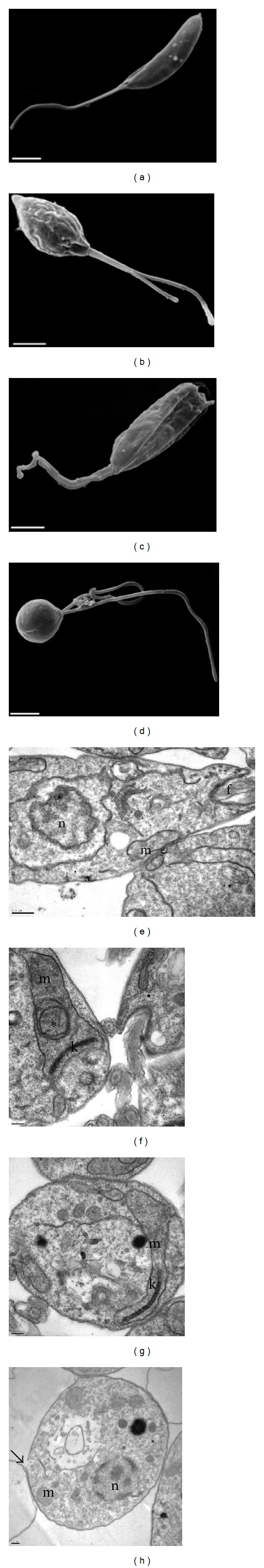
Scanning electron microscopy (SEM) and transmission electron microscopy (TEM) of *Leishmania amazonensis* promastigotes after 48 h of treatment with **C4**. (a) SEM image of an untreated promastigote, showing typical elongated morphology. (b) and (c) SEM images of promastigotes after treatment with the IC_50_ (16.0 *μ*M), showing distortion of the cell body. (d) SEM image of promastigote after treatment with the IC_90_ (103.0 *μ*M), showing rounding of the cell body. Scale bar: 2 *μ*m. (e) TEM image of untreated promastigote, showing normal mitochondria (m), nucleus (n), and flagellum (f). (f) and (g) TEM images of promastigotes treated with **C4** at 16.0 *μ*M, showing mitochondrial swelling and the presence of concentric membranes inside mitochondria (black asterisk). (h) Promastigotes treated with **C4** at 103.0 *μ*M, showing alteration of the plasma membrane (black arrow). Scale bar: 0.5 *μ*m in (e) and 0.2 *μ*m in (f)–(h).

Morphological alterations in promastigotes treated with **C4** were observed using scanning electron microscopy. The control showed normal characteristics of the parasite, such as an elongated body and free flagellum (Figure 2(a)). Parasites treated with the IC_50_ of **C4** exhibited distortions in the cell body (Figures 2(b) and 2(c)), and the group treated with the IC_90_ showed rounding of the cell body (Figure 2(d)).

Ultrastructural changes in promastigotes treated with **C4** at the IC_50_ and IC_90_ are illustrated in Figure 2, showing significant alterations. The mitochondria showed intense swelling (Figures 2(f) and 2(g)), the presence of concentric membrane structures inside the organelle (Figure 2(f)), and alterations of the plasma membrane (Figure 2(h)). These ultrastructural changes were not observed in untreated parasites (Figure 2(e)).

### 3.4. Mitochondrial Membrane Potential

**Figure 3:**
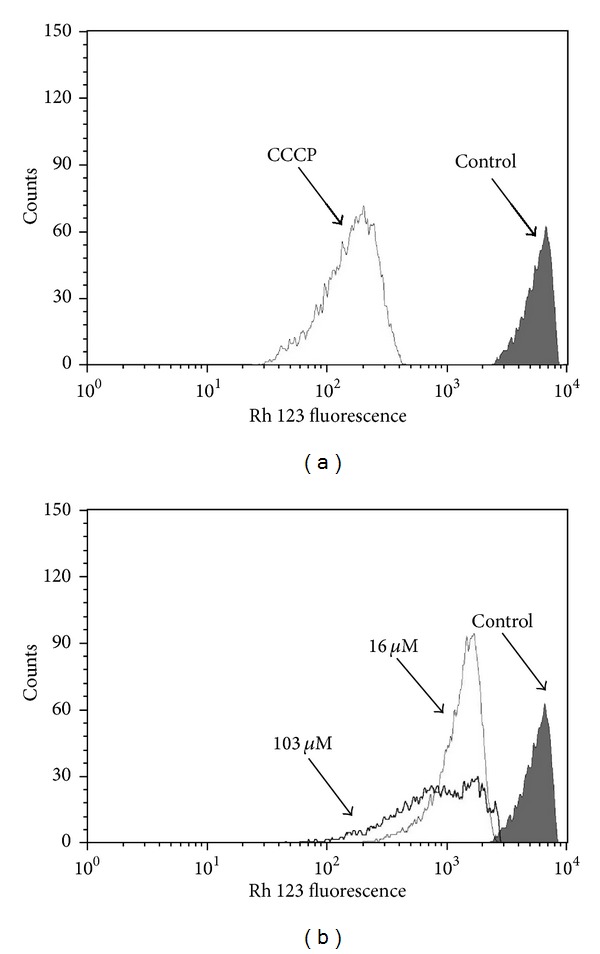
Flow cytometry analysis of promastigotes of *Leishmania amazonensis* treated with **C4** for 24 h and stained with Rh 123. (a) Promastigotes treated with 100 *μ*M CCCP (positive control). (b) Promastigotes treated with 16.0 and 103.0 *μ*M of **C4**. The control group (i.e., untreated parasites) is also shown.

The effect of **C4** on mitochondrial membrane potential (ΔΨm) in promastigote forms was assessed by flow cytometry using Rh 123, a fluorescent marker that indicates mitochondrial membrane potential. The IC_50_ and IC_90_ induced 73.3% and 82.5% decreases in total Rh 123 fluorescence intensity, respectively, compared with the control group, indicating depolarization of mitochondrial membrane potential (Figure 3(b)). Promastigotes treated with CCCP showed a 94.8% decrease in membrane potential (Figure 3(a)).

### 3.5. Plasma Membrane Integrity

**Figure 4:**
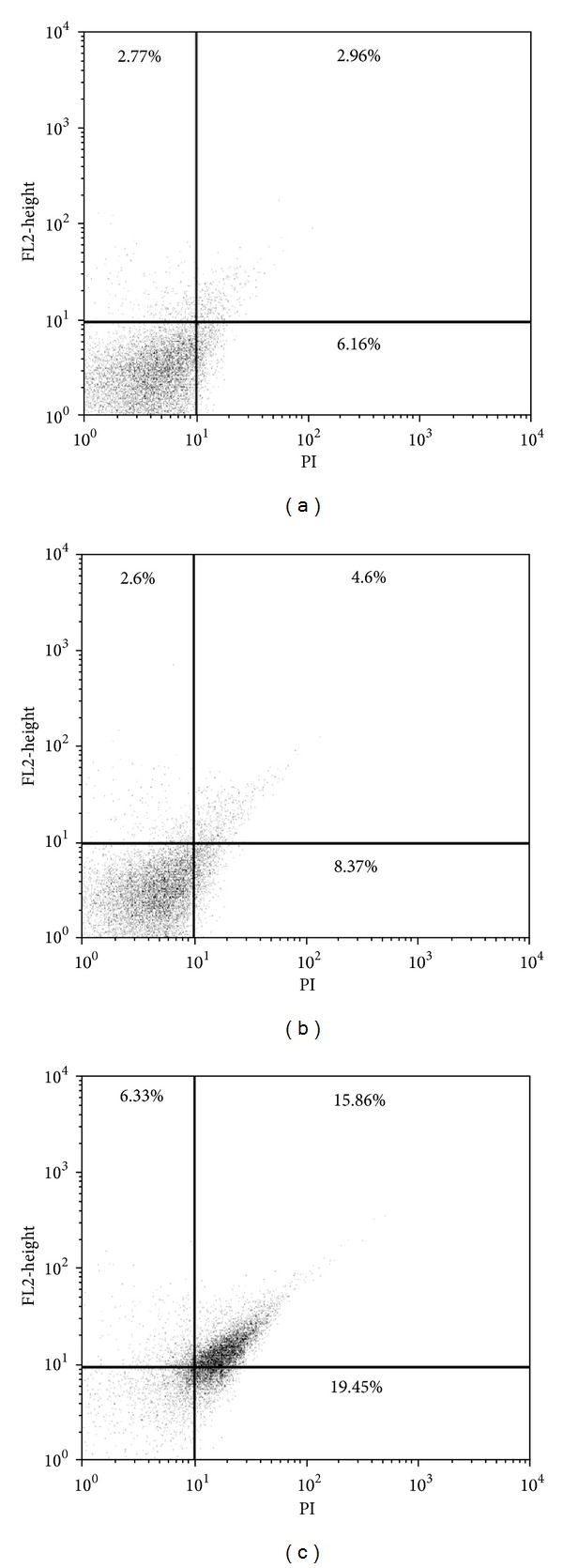
Flow cytometry analysis of promastigotes of *Leishmania amazonensis* treated with **C4** for 24 h and stained with propidium iodide (PI). (a) Control group (i.e., untreated cells). (b) Promastigotes treated with 16.0 *μ*M of **C4**. (c) Promastigotes treated with 103.0 *μ*M of **C4**. The percentages of PI-stained positive cells are shown in the upper right and left quadrants.

Cell membrane integrity in promastigotes treated with **C4** was assessed by flow cytometry using PI, which diffuses across disrupted plasma membranes of cells and binds to nucleic acids. **C4** at the IC_90_ increased total PI fluorescence intensity by 22.2% (Figure 4(c)), compared with the control group (5.7%; Figure 4(a)), indicating the alteration of cell membrane integrity. However, **C4** at the IC_50_ did not induce any membrane alterations (Figure 4(b)). The positive control (i.e., digitonin) also showed an increase in fluorescence (data not shown).

### 3.6. Detection of Mitochondria-Derived O_2_^∙−^

**Figure 5:**
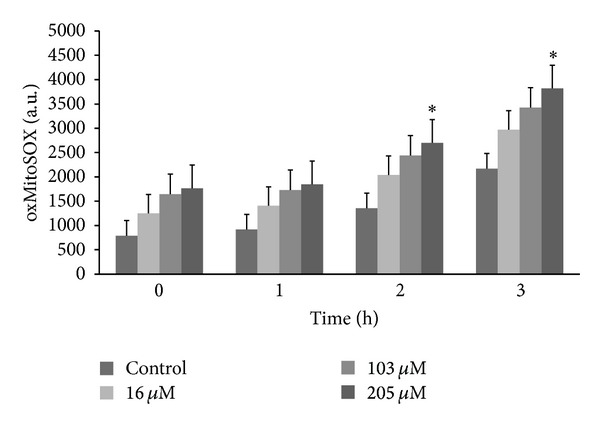
Mitochondrial O_2_^∙−^ production in promastigote forms of *Leishmania amazonensis* treated with **C4** for up to 3 h. Mitochondrial O_2_^∙−^ production was evaluated using the fluorescent probe MitoSOX. At the indicated times, promastigotes were used to measure oxidized MitoSOX (oxMitoSOX). The results are expressed in arbitrary units (mean ± SE of at least three independent experiments). *P ≤ 0.05, significant difference compared with the control group (i.e., untreated cells; two-way analysis of variance followed by Tukey post hoc test).

The production of O_2_^∙−^ was evaluated in promastigotes treated with **C4** using MitoSOX reagent, which measures the accumulation of mitochondrial superoxide.  Figure 5 shows that **C4** increased the production of mitochondrial O_2_^∙−^ at all concentrations and times tested compared with the control group. However, **C4** induced a significant increase in O_2_^∙−^ production only after 2 and 3 h of treatment at the higher concentration (205 *μ*M). The positive control (i.e., antimycin A) also showed an increase in fluorescence (data not shown).

## 4. Discussion

Several natural and synthetic compounds have been studied for the treatment of leishmaniasis [22–29]. Nevertheless, the treatment of this infection remains a problem because of the high toxicity and adverse side effects. *β*-Carbolines have been previously reported to have various biological properties, including antioxidant, antimicrobial [30], antiparasitic [12, 13], antitumoral [31], and antiviral effects [32]. These compounds also exhibited activity against protozoa, such as *T. cruzi*, especially against bloodstream trypomastigotes. Our previous studies demonstrated the effective and selective action of **C4** against *T. cruzi* [12, 13]. The present study evaluated the antileishmanial activity of **C4** against *L. amazonensis *and its possible targets in this parasite.

**C4** compound inhibited the growth of the promastigote forms of the parasite and caused morphological and ultrastructural alterations, especially in mitochondria. The Rh 123 assay confirmed the mitochondrial action of **C4**, reflected by a decrease in Rh 123 fluorescence intensity. The mitochondria of trypanosomatids are attractive chemotherapeutic targets because they have structural and functional characteristics that are distinct from mammalian mitochondria [33]. In fact, increasing reports have described compounds that target parasite mitochondrial destabilization and disorganization [24, 26].

Mitochondrial alterations may be a consequence of many potentially harmful effects induced by both exogenous and endogenous toxic compounds. We currently consider **C4** as an exogenous toxic compound. However, **C4** may also induce the production of endogenous toxic compounds, including reactive oxygen species (ROS), that may be responsible for mitochondrial dysfunction and induce oxidative damage in lipids and proteins, the main macromolecules of biological membranes. This indirect effect of **C4** was demonstrated in the MitoSOX and PI assays in the present study, similar to the effects of compound reported by Desoti et al. [34].

In conclusion, our data indicate that **C4** is a leishmanicidal compound that is able to induce parasite disorders that are mainly mediated by mitochondrial dysfunction. Further *in vitro* and *in vivo* studies are necessary to increase our understanding of the mode of action of this compound and determine whether it can be exploited alone or in combination with other drugs for the treatment of antileishmaniasis. 

## Figures and Tables

**Table 1:** Effect of **C4** against promastigote and axenic amastigote forms of *Leishmania amazonensis*, cytotoxicity in macrophages cells, and selectivity index.

Cells	**C4** (*µ*M) IC_50_^a^	**C4** (*µ*M) IC_90_^b^	SI^c^
Promastigotes	16.0 ± 2.28	103.0 ± 20.17	45.1
Axenic amastigotes	16.3 ± 2.38	118.0 ± 45	44.2
Macrophages (CC_50_)^d^	722.0 ± 45	—	—

^a^Concentration that inhibited 50% of growth.

^b^Concentration that inhibited 90% of growth.

^c^Selectivity index (SI; CC_50_ macrophages/IC_50_).

^d^50% cytotoxicity concentration in macrophages.
